# Exploring implementation barriers of mental health programs in primary health care: a qualitative study from Iran

**DOI:** 10.1186/s12913-025-13702-9

**Published:** 2025-11-28

**Authors:** Mehrdad Salehi, Fahimeh Hormozi, Aboalfazl Marvi, Bita Sedigh, Abdol Jabbar Zakeri

**Affiliations:** 1 Social Determinants in Health Promotion Research Center, Hormozgan Health Institute, Hormozgan University of Medical Sciences, Bandar Abbas, Iran; 2 Student Research Committee, Mashhad University of Medical Sciences, Mashhad, Iran; 3 Department of Community Medicine, Faculty of Medicine, Hormozgan University of Medical Sciences, Bandar Abbas, Iran

**Keywords:** Mental health, Primary health care, Implementation barriers, Qualitative study, Iran

## Abstract

**Background:**

Integrating mental health services into primary health care (PHC) has been globally recognized as a key strategy to improve access, reduce treatment costs, and enhance the continuity of care—especially in low- and middle-income countries (LMICs). Despite substantial policy efforts in Iran, the effective implementation of these programs remains suboptimal, with limited understanding of the underlying barriers from the perspective of healthcare professionals. This study aimed to explore and analyze the barriers to implementing mental health programs within Iran’s PHC system, drawing on the experiences and insights of frontline health professionals.

**Methods:**

This qualitative study was conducted in 2024 in Hormozgan Province, southern Iran, using a conventional content analysis approach. Data were collected through 16 in-depth, semi-structured interviews with psychologists, community health workers, and mental health officers. Interviews were transcribed verbatim and analyzed using MAXQDA software, following the principles of inductive coding and thematic categorization.

**Results:**

Four main categories of implementation barriers were identified: (1) human and organizational capacity limitations (ineffective inter-professional collaboration, lack of training, and excessive workload) (2), infrastructural and environmental deficiencies (insufficient counseling spaces, poor accessibility, and inadequate technical resources) (3), cultural and social barriers (stigma, low public awareness, and resistance from patients or families), and (4) institutional and systemic challenges (program overload, weak data systems, unrealistic performance indicators, and limited inter-sectoral coordination).

**Conclusion:**

This study highlights critical human, infrastructural, cultural, and institutional barriers that hinder the effective integration of mental health services into PHC in Iran. Strengthening workforce capacity, improving physical and digital infrastructure, promoting community awareness, and reforming performance and governance systems are essential steps toward sustainable implementation.

**Clinical trial registration:**

Not applicable.

## Introduction

In recent decades, mental health has emerged as a central priority in global health systems and policy agendas. According to the World Health Organization (WHO), approximately one in eight people worldwide suffers from a mental disorder [1]. These disorders, ranging from depression and anxiety to severe conditions such as schizophrenia, not only impair individual and familial quality of life but also represent major contributors to reduced economic productivity, increased disability, and escalating health expenditures at both national and global levels [2, 3]. In low- and middle-income countries (LMICs) in particular, the burden of mental disorders is exacerbated by factors such as rapid urbanization, poverty, social inequality, and recurrent humanitarian crises [4, 5].

Despite the growing demand for care, there remains a substantial gap between mental health needs and access to effective services. It is estimated that over 75% of individuals with mental health conditions in LMICs receive no appropriate treatment [6]. To address this crisis, the WHO and other international bodies have advocated for the integration of mental health services into primary health care (PHC) systems [7, 8]. This approach, which aligns with the Universal Health Coverage (UHC) model, seeks to ensure accessible, affordable, non-discriminatory, and continuous mental health services for all segments of the population [9, 10].

As the first point of contact with the health system, PHC presents a unique opportunity for early identification of mental disorders, delivery of non-specialist interventions, public education, and follow-up care [11]. Evidence from various countries indicates that integrating mental health services into PHC can lower treatment costs, reduce stigma and discrimination, and improve clinical outcomes [12–14]. In this context, tools such as the WHO’s Mental Health Gap Action Programme (mhGAP) have been developed to enable the provision of basic mental health care by non-specialist providers within PHC systems [15].

Despite the strong global evidence base, the successful implementation of integrated mental health services at the PHC level is often hindered by multiple barriers. These include the absence of stable and coordinated mental health policies, a shortage of trained human resources, insufficient funding, weak health information systems, social stigma, limited intersectoral collaboration, and cultural and linguistic challenges [16–19]. In many countries, PHC staff not only lack adequate mental health training but also face high workloads, limited motivation, and insufficient institutional support [20, 21].

In Iran, considerable efforts have been made over the past two decades to expand mental health services within PHC. These include the establishment of community-based mental health centers, development of essential mental health service packages within the health network system, and implementation of innovative intervention models such as the rural mental health model [22]. However, challenges remain in ensuring service coverage, continuity of care, community engagement, and the overall quality of mental health service delivery [23]. In many regions, particularly underserved provinces, access to specialized mental health services is severely limited. The heavy reliance on multipurpose health workers without adequate training further compromises service quality [20].

A key gap in the existing literature in Iran is the lack of qualitative and field-based studies exploring the perspectives of healthcare personnel. While these individuals play a crucial role in the implementation of mental health programs, they are rarely involved in policy design, evaluation, or revision. A deeper understanding of their experiences, perceptions, and the barriers they encounter in practice could inform the development of more realistic and actionable strategies.

Despite the global emphasis on evidence-based policymaking, there remains a shortage of qualitative research exploring the contextual realities of implementing mental health programs in PHC systems, particularly in low- and middle-income countries such as Iran. Quantitative indicators alone are insufficient to capture the complex social, organizational, and cultural factors that influence program delivery. Qualitative inquiry provides unique insights into the lived experiences, perceptions, and interactions of frontline health workers, allowing for a deeper understanding of implementation dynamics that are often overlooked in numerical data. Previous Iranian studies have mostly focused on service outcomes or policy analysis [20, 22, 23], while few have examined barriers from the practitioners’ standpoint. Previous Iranian qualitative studies have also emphasized the importance of exploring professionals’ competencies and contextual barriers in health program implementation, particularly in areas such as disaster management and clinical practice improvement [24, 25]. International research also highlights that qualitative approaches are critical in revealing hidden systemic gaps and in shaping context-sensitive interventions [16, 18, 26, 27]. Therefore, adopting a qualitative design in this study enables a more comprehensive and realistic assessment of the challenges faced in implementing mental health programs within primary health care.

This study was designed to identify and analyze the implementation barriers of mental health programs at the PHC level in Iran from the perspective of frontline healthcare workers. The findings aim to shed light on structural, managerial, social, and professional challenges, thereby contributing to more effective policy-making and improved implementation of mental health services within the national PHC framework.

## Methods

### Study design

This study employed a qualitative design using the conventional content analysis approach. The study was conducted in 2024 in primary health care (PHC) centers located in Hormozgan Province, southern Iran. This design was chosen to provide an in-depth understanding of the barriers to implementing mental health programs from the perspective of frontline healthcare professionals.

### Participants and sampling

Participants in this study were selected using a purposive sampling strategy, which allows the researcher to intentionally recruit individuals who possess firsthand knowledge and experience relevant to the research objectives [28]. Eligible participants were those who had at least three years of experience in the field of mental health within primary health care (PHC) settings and who were both willing and available to participate in the interviews. To ensure a diverse representation of viewpoints, the sample included professionals with different occupational roles—such as psychologists, community health workers, and mental health officers—from both urban and rural health centers. This approach was designed to capture variations in perspectives arising from different service contexts and professional responsibilities.

Sampling continued until data saturation was achieved, meaning that no new codes or themes were emerging from additional interviews. Saturation occurred after the thirteenth interview, and three further interviews were conducted to confirm the completeness and adequacy of the data [29]. The purposive and iterative nature of this process enhanced the credibility and trustworthiness of the findings by ensuring alignment between participant characteristics and the goals of the study [30].

### Data collection

Data were collected through semi-structured, face-to-face interviews conducted over two months (November–December 2024). Each interview lasted between 30 and 60 min and was conducted in a quiet, private room within the participants’ respective health centers to ensure comfort and confidentiality. The initial interview guide was developed based on a brief review of existing literature and through consultation with the research team. Following three pilot interviews, minor adjustments were made to improve clarity and flow. The interview guide included both general and open-ended questions, supported by probing items for clarification (Table 1). Before the start of each interview, the researcher explained the aim and significance of the study to participants to foster trust and openness. Written informed consent was obtained, and participants were reassured that their responses would remain anonymous and confidential. Interviews were conducted by a researcher with a Master’s degree in Health Services Management (FH), who had prior experience in PHC service delivery. All interviews were audio-recorded with participants’ permission, and non-verbal cues were documented in field notes. Transcriptions were prepared immediately after each session, and early analyses informed subsequent interviews.

**Table 1:** Interview guide questions

No.	Question
1	Please provide a brief self-introduction.
2	What is your highest academic qualification?
3	How many years of professional experience do you have in the field of mental health?
4	How would you describe the process of implementing mental health programs in your work setting?
5	In your opinion, what are the main barriers and challenges to the effective implementation of mental health services (including prevention, screening, diagnosis, care, and follow-up) at the primary healthcare level in Hormozgan Province?
6	How would you assess the current status of mental health indicators in your setting? If they do not meet expectations, what are the underlying reasons?

Note for Interviewers:

Follow-up and probing questions (such as *“Could you elaborate further?”*, *“Can you provide an example?”*, or *“What do you mean by that?”*) may be used during the interview to obtain deeper insights and clarifications

### Data analysis

Data analysis followed the conventional content analysis framework [31]. Transcribed interviews and observational notes were entered into MAXQDA (version 20) software [32] for systematic coding. The analysis proceeded concurrently with data collection. Researchers (AM and MS) independently performed line-by-line coding to extract meaning units, which were then condensed and grouped into codes, subcategories, and main categories based on semantic similarity. All emerging themes were discussed and refined in team meetings to ensure conceptual coherence and analytical depth.

### Trustworthiness

To ensure the rigor and trustworthiness of the findings, the study adhered to the four criteria proposed by Guba and Lincoln—credibility, dependability, confirmability, and transferability [33]. Credibility was strengthened by employing purposive sampling with maximum variation, allowing the inclusion of participants with diverse professional roles and experiences within PHC settings. The researchers maintained continuous engagement throughout data collection and analysis to ensure a deep understanding of participants’ perspectives. Dependability was reinforced through *member checking*, where transcripts and preliminary interpretations were returned to participants for validation, as well as by conducting dual coding independently by two researchers to ensure analytical consistency. Confirmability was achieved by maintaining an audit trail of analytic decisions and engaging the entire research team in reviewing and confirming the emerging categories and themes, thereby minimizing researcher bias. Finally, transferability was enhanced by providing a rich, detailed description of the study context, sampling strategy, and participant quotations, enabling readers to assess the applicability of the findings to similar settings.

### Ethical approval and consent for participation

Ethical approval for this study was obtained from the Ethics Committee of Hormozgan University of Medical Sciences (IR.HUMS.REC.1402.374). Participants were informed orally and in writing about the study objectives, voluntary participation, and confidentiality of their information. The ethical principles of autonomy, non-maleficence, privacy, and data protection were strictly observed, in accordance with the Declaration of Helsinki [34].

### Study limitations

This qualitative study was limited to a specific province and a defined group of PHC providers, which may influence the transferability of findings to other contexts. Moreover, qualitative research seeks depth of understanding rather than statistical generalizability. However, the methodological rigor and diversity of participants enhance the credibility and contextual richness of the results.

## Results

Table 2 presents the demographic characteristics of the interview participants. According to the findings, the mean age of participants was 28.1 years, and the average duration of work experience related to mental health was 6.4 years. Among the participants, 66.6% were female, and 77.7% held a bachelor’s degree (Table 2).

Table 3 outlines the key themes, categories, and subcategories derived from the data analysis. The barriers and challenges to implementing mental health programs within primary health care, as reported by participants, were organized into four main categories: *human and organizational capacity limitations*, *infrastructural and environmental deficiencies*, *cultural and social barriers*, and *institutional and systemic challenges*. These overarching categories encompass a total of 13 subcategories (Table 3).

**Table 2:** Demographic information of the study participants

Variable		*N*	%
Gender	Male	6	33.3
	Female	12	66.6
Academic degree	MSc in Psychology	4	22.2
	Bachelor	14	77.7
Positions	psychologist	8	44.4
	Community health workers	10	55.5
Average age(years)	28.1		
Job experiences (years)	6.4		

**Table 3:** Barriers to mental health program implementation

Category	Subcategory	Items
Human and Organizational Capacity Limitations	Ineffective inter-professional collaboration	• Lack of effective interaction between mental health providers and general practitioners • Physicians’ distrust in the effectiveness of the mental health registration system • Physician demotivation
	Lack of specialized skills and training	• Inadequate staff skills • Lack of structured training programs • Reduced training opportunities post-COVID-19 pandemic • Shortage of specialized training
	Workload and staff shortages	• High workload • Shortage of human resources • Occupational fatigue
Infrastructure and Environmental Deficiencies	Lack of proper space and privacy	• Inadequate physical condition of counseling rooms • Patients’ reluctance to discuss issues openly
	Inadequate physical access and low safety	• Inappropriate physical design of health centers for elderly • Female staff’s sense of insecurity • Absence of halls and educational facilities for group sessions
	Technical and equipment limitations	• Frequent internet disconnections • Lack of telephones
Cultural and Social Barriers	Stigma and negative attitudes towards mental disorders	• Public’s negative perception of mental health problems • Fear of judgment • Social shame • Denial of the problem
	Lack of public awareness of mental health services	• Lack of awareness about free mental health services • Preference for private consultants • Scarcity of awareness and education campaigns • Deliberate resistance from patients or their families • Concern over stigma or disclosure of family issues • Disruption in treatment process and reduced effectiveness of interventions
Institutional and Systemic Challenges	Program overload and role multiplicity	• Excessive number of parallel programs • Seasonal shifts in work priorities
	Weak screening tools and uncoordinated registration systems	• Inadequate screening tools • Misalignment of screening tools with clinical protocols • Lack of coordination between electronic health records and protocols • Removal of key system functions such as automated SMS notification • Lack of coordination and effective interaction with related institutions
	Unrealistic indicators and performance monitoring limitations	• Mismatch of performance indicators with operational realities • Lack of transparent demographic data • Absence of periodic reports on indicator achievement

### Main Theme 1: Human and organizational capacity limitations

Findings revealed that one of the key structural barriers to the effective implementation of mental health programs in primary health care (PHC) centers was the weakness in human and organizational capacity. This theme comprised three interrelated subcategories: **ineffective inter-professional collaboration**, **lack of specialized skills and training**, and **high workload and staff shortages**. Together, these elements reflect how limited professional support, inadequate preparation, and overwhelming responsibilities hinder the quality and continuity of mental health service delivery.

#### Ineffective inter-professional collaboration

Participants emphasized that insufficient communication and collaboration between general practitioners and mental health workers significantly disrupted the delivery of integrated care. Physicians often showed reluctance to diagnose or manage mental health conditions, viewing such responsibilities as outside their professional scope. This limited engagement not only delayed clinical decisions but also led to ambiguity in follow-up and referrals. One participant explained that *“Physicians usually don’t prescribe medications or even make diagnoses*,* so I either refer the patient or try to convince the physician to make a diagnosis”* (Interviewees 3, 11, and 15). Another added, *“The physician doesn’t see it as their role to register the condition*,* but most of my work depends on them because they are the ones who must register the disorder”* (Interviewees 13 and 14). In several cases, physicians’ distrust in the health information system further weakened collaboration. As one participant reported, *“A doctor once said*,* ‘Why should I register the diagnosis? Our health system doesn’t do anything with it. The system is weak’”* (Interviewee 8). Consequently, health workers were often compelled to assume responsibilities beyond their official roles to ensure patient care continuity.

**Lack of specialized skills and training** The second major barrier identified was the inadequate preparation of health personnel for addressing mental health problems. Many participants noted that some staff were assigned to mental health duties without prior experience or targeted training, which reduced their confidence and performance quality. One participant explained, *“We had contract workers who weren’t suitable for mental health work and lacked the necessary skills”* (Interviewee 5). This limitation was especially evident in handling complex or sensitive cases involving psychiatric or substance use disorders. Additionally, participants reported that in-service training opportunities had sharply declined after the COVID-19 pandemic: *“Group training sessions should be held more often; we used to have many before COVID*,* but they stopped”* (Interviewee 9). Some participants also highlighted gaps in the training content itself, such as the lack of instruction on sexual health or communication with high-risk clients. As one interviewee stated, *“There are limitations in what is taught—sexual education*,* for example*,* is not included in training; I had to add it myself”* (Interviewee 15). These findings underline the need for systematic and continuous capacity-building programs for PHC personnel.

**High workload and staff shortages** The third subcategory addressed the pervasive problem of excessive workload and inadequate staffing. The coexistence of multiple parallel health programs placed heavy administrative and clinical demands on PHC workers, often resulting in fatigue, stress, and reduced service quality. As described by one health worker, *“There’s too much work for health providers; screening and follow-up have decreased”* (Interviewee 1). Another remarked, *“There are too many programs for health workers; recently*,* traditional medicine was added too—some screenings aren’t done properly anymore”* (Interviewee 14). Participants also mentioned that the absence of substitutes during leave periods created service disruptions, particularly for vulnerable clients: *“When we go on leave*,* some social or addiction cases get missed”* (Interviewee 2). Furthermore, the irregular attendance of psychologists and psychiatrists at the centers negatively affected the continuity of care. As one participant observed, *“If the psychiatrist or psychologist were present every day*,* people would be more likely to accept mental health services”* (Interviewee 16).

Overall, the absence of structured workforce development, weak inter-professional collaboration, and the cumulative pressure of excessive workload emerged as major obstacles to effective mental health service delivery in PHC settings. Strengthening teamwork, providing ongoing technical training, and ensuring adequate staffing are essential prerequisites for the sustainable integration of mental health programs within primary care.

### Main Theme 2: Infrastructural and environmental deficiencies

Another major barrier to the effective delivery of mental health services in primary health care (PHC) centers was the set of limitations related to physical, technical, and environmental infrastructure. This theme comprised three interconnected subcategories: **inadequate counseling space and lack of privacy**, **poor physical accessibility and safety concerns**, and **technical limitations and insufficient administrative equipment**. Collectively, these barriers illustrate how the material and structural context of PHC facilities can directly influence the quality, confidentiality, and continuity of mental health care.

#### Inadequate counseling space and lack of privacy

Participants consistently highlighted the unsuitability of counseling rooms for private and confidential communication. Many reported that available spaces were shared with other staff members, leading to interruptions and discomfort for clients. Such conditions undermined clients’ willingness to disclose sensitive personal information, particularly regarding domestic issues or mental distress. One participant stated, *“The infrastructure of our center is not standard; even the receptionist and doctor can hear the conversations. Clients need to feel secure”* (Interviewee 5). Another added, *“I share a room with the nutritionist. During consultations*,* we take turns leaving the room”* (Interviewees 8 and 11). The shared and crowded environment often made mental health screenings difficult and inconsistent. As one participant explained, *“Screening has become difficult because several health workers are working in the same room”* (Interviewee 4). In such non-private settings, patients often avoided discussing highly sensitive issues. One interviewee emphasized, *“Clients need to feel comfortable enough to talk about themselves*,* their husbands*,* and their problems… I haven’t seen any center that offers such a space”* (Interviewee 7). These accounts reveal a critical gap between the psychological safety required for counseling and the actual environmental conditions in many PHC centers.

**Poor physical accessibility and safety concerns**: Another infrastructural barrier involved the physical layout and security conditions of PHC centers, which affected both service accessibility and staff safety. The design of many buildings posed challenges for older adults and people with mobility difficulties. As one participant described, *“There are 18–19 steps to reach the center; most of our clients are older adults”* (Interviewees 14 and 5). Additionally, the absence of male security personnel in some facilities caused discomfort among female staff, particularly when managing difficult or aggressive clients. *“Some male clients make us feel unsafe because there is no male guard”* (Interviewee 5). Participants also referred to the lack of dedicated spaces for group education or psychoeducation sessions as a missed opportunity for community engagement: *“We don’t have a hall for education sessions where we can show educational films using a projector”* (Interviewee 8). The absence of such facilities limited the centers’ ability to provide family or group-based interventions, which are essential components of community mental health programs.

**Technical limitations and insufficient administrative equipment** A third subcategory of infrastructural challenges related to the lack of reliable digital and administrative resources. Frequent internet disconnections, limited access to telecommunication tools, and shortages of essential materials such as follow-up booklets were commonly cited. One participant mentioned, *“We often have internet disruptions or very weak connections”* (Interviewee 2). Another reported, *“There is only one telephone in the room*,* and four people have to share it”* (Interviewee 3). Others expressed frustration with the lack of basic materials: *“We need booklets for follow-up*,* but we’re told to provide them ourselves”* (Interviewee 10), and *“There is a shortage of equipment—our center has new health workers*,* but they don’t even have desks or computers”* (Interviewee 13). These operational deficiencies not only slowed routine work but also reduced the efficiency of case management and data documentation.

Overall, infrastructural and environmental limitations—ranging from the lack of private spaces and accessibility challenges to inadequate technical resources—represent significant obstacles to the consistent and high-quality implementation of mental health services in PHC settings. Addressing these deficiencies through investment in physical infrastructure, digital tools, and workplace safety can substantially improve both staff performance and patient trust.

### Main Theme 3: Cultural and social barriers

The analysis of the interviews revealed that prevailing cultural and social attitudes in the community constitute some of the most deep-rooted barriers to the identification, acceptance, and follow-up of mental health disorders within the primary health care (PHC) system. This theme encompassed three key subcategories: **stigma and negative perceptions of mental illness**, **public unawareness of available services**, and **lack of cooperation from patients and families**. Together, these findings highlight how cultural beliefs, social norms, and interpersonal dynamics influence individuals’ willingness to seek and sustain mental health care.

#### Stigma and negative perceptions of mental disorders

Many participants emphasized that stigmatizing attitudes toward mental health problems discourage individuals from seeking services at PHC centers. Fear of judgment, social shame, and denial of illness were frequently cited as reasons for non-engagement with the health system. Participants explained that social labeling of mental illness as a “disgrace” or a “family shame” often silenced discussion and delayed care-seeking. As expressed by participants, *“People consider mental illness a disgrace—they don’t talk about it”* (Interviewee 1), and *“Negative social perceptions about mental disorders make people avoid consulting mental health professionals”* (Interviewee 14). Several respondents also mentioned dishonesty during screening sessions as a manifestation of this stigma. For example, *“During addiction screening*,* no one tells the truth”* (Interviewee 3), and *“People care more about physical health than mental health”* (Interviewee 7). These narratives indicate that pervasive stigma not only limits acknowledgment of illness but also disrupts effective screening, diagnosis, and long-term follow-up.

**Public unawareness of mental health services** Another major social barrier identified in this study was the lack of awareness about free mental health services offered at PHC centers. Many community members either remained unaware of these services or perceived mental health care as something accessible only through expensive private clinics. As one participant noted, *“People spend a lot of money on private consultations*,* but they don’t know services here are free”* (Interviewee 3). Participants believed that improved communication and public engagement could bridge this gap. Several practical strategies were suggested, such as the use of SMS notifications, community-based signage, and media campaigns: *“We used to send text messages for vaccines—we should do the same for mental health”* (Interviewee 5); *“There is no mention of mental health in the media”* (Interviewee 7); and *“If we put up banners and signs in neighborhoods*,* it would help”* (Interviewee 6). Others emphasized the importance of outreach programs and awareness campaigns to promote mental health literacy: *“We should have mental health campaigns*,* just like the physicians and health workers do for other issues—maybe not door-to-door*,* but something similar for case identification”* (Interviewee 12). These perspectives underline the urgent need for creative, culturally sensitive communication strategies to increase public understanding and normalize help-seeking behavior.

**Lack of cooperation from patients and families** Beyond limited awareness, some participants described active resistance from patients or their families to engage with psychological services. This resistance was often linked to concerns about stigma, gossip, or the disclosure of private family matters. Such fears led to delayed or disrupted treatment processes, especially in sensitive cases such as suicide attempts or substance use. One participant shared, *“We had a suicide case*,* and the family said: ‘Leave it*,* don’t tell anyone’—which was very difficult”* (Interviewee 16). Others observed that some individuals avoided therapy despite its accessibility and low cost: *“Some patients don’t cooperate*,* even though the services are free; it’s related to their mindset”* (Interviewee 6). A similar pattern was reported among adolescents, who were often reluctant to attend sessions: *“Teenagers need counseling*,* but they don’t come to the health center”* (Interviewee 5). Such resistance—particularly among youth and women facing psychosocial stressors—significantly undermines the continuity and effectiveness of care.

In summary, these findings illustrate a **cultural–social triangle** consisting of stigma, limited public awareness, and family-level resistance that collectively hinders the expansion and normalization of mental health services within PHC settings. Addressing these barriers requires community-based education, collaboration with local opinion leaders, and media engagement to foster acceptance and understanding of mental health as an integral component of overall well-being.

### Main Theme 4: Institutional and systemic challenges

In addition to individual, social, and environmental barriers, the findings of this study revealed that **structural inefficiencies at the macro level of the health system** play a major role in undermining the quality and consistency of mental health program implementation in primary health care (PHC) centers. This theme comprised four interrelated subcategories: **program overload and task duplication**, **weaknesses in screening tools and registration systems**, **lack of inter-sectoral collaboration**, and **unrealistic performance indicators with monitoring limitations**. Together, these challenges reflect deeper institutional problems that limit the sustainability and scalability of mental health initiatives within the PHC framework.

#### Program overload and task duplication

Participants described how the simultaneous implementation of numerous national health programs reduced their capacity to focus on mental health-related activities. Periodic campaigns—such as immunization drives, chronic disease screenings, and health promotion programs—often took precedence over psychological services, leading to fragmented care delivery. One participant explained, *“In the winter*,* programs like supplementary polio immunization and diabetes screening take up most of our time”* (Interviewee 8). Another added, *“Due to the high workload*,* mental health screenings are not performed thoroughly”* (Interviewee 4). The overlap of multiple tasks, combined with persistent staff shortages, resulted in fatigue, reduced motivation, and a noticeable decline in the quality of counseling and follow-up. These conditions contributed to a sense of inefficacy among health workers, undermining the continuity of mental health interventions.

**Weaknesses in screening tools and registration systems** Another frequently mentioned challenge was the inadequacy of the screening instruments and data management systems used in PHC. Participants noted that the tools for identifying mental health disorders were overly simplistic and poorly aligned with clinical guidelines, limiting their diagnostic value. As one participant emphasized, *“We need more questions to assess depression—one positive response is not enough”* (Interviewee 1). Additionally, several interviewees expressed frustration with the integrated electronic health system (SIB), describing it as incompatible with clinical workflows and insufficiently responsive to operational needs. *“The SIB system is not aligned with protocols; the connection doesn’t work properly”* (Interviewee 4). The removal of automated features such as SMS reminders was also reported to increase workload and reduce patient adherence: *“We used to send reminders through the system*,* but that feature no longer exists”* (Interviewee 9). These system inefficiencies not only delayed follow-up but also reduced the accuracy of reporting and monitoring activities.

**Lack of inter-sectoral collaboration** Participants repeatedly highlighted the absence of effective coordination between the health system and other key sectors such as education, hospitals, and social services. This lack of integration was particularly evident in cases requiring multidisciplinary intervention, such as suicide prevention or substance use management. As one participant described, *“Hospital referrals for suicide cases are very weak”* (Interviewee 4). Another reported resistance from schools and parents during mental health screening efforts: *“Schools and parents resisted the suicide screening program*,* and it failed”* (Interviewee 6). Such fragmentation prevents information sharing and disrupts the continuity of care, ultimately weakening the overall effectiveness of mental health programs. Strengthening inter-sectoral linkages is therefore essential for building a more cohesive mental health delivery network.

**Unrealistic performance indicators and monitoring limitations** A fourth systemic barrier involved the use of quantitative performance indicators that were poorly matched with on-the-ground realities. Participants described these metrics as demotivating and unachievable, creating administrative pressure rather than facilitating quality improvement. As expressed by participants, *“The target of 12% positive screenings doesn’t match our client volume—I work all day but still fall short”* (Interviewee 6), and *“The requirement for 80 initial mental health visits per month is too much—we can’t meet it”* (Interviewee 6). Many interviewees also believed that mental health remained a low institutional priority compared to other health programs: *“Our system hasn’t really prioritized mental health”* (Interviewee 14). Unreliable population data and a lack of regular performance feedback further limited local decision-making capacity. One participant noted, *“We’re supposed to cover 40*,*000 people*,* but it’s unclear how many actually live here”* (Interviewee 8), while another added, *“We provide services to temporary visitors*,* but they don’t count in the indicators”* (Interviewee 13). Some participants also described confusion and inefficiency in follow-up processes: *“Sometimes I identify a case and tell the health worker it’s positive*,* but some of them fall through the cracks”* (Interviewee 10), and *“We share a room with two staff; the health worker’s tasks are scattered*,* and follow-up for identified cases gets lost in the process”* (Interviewee 11). These accounts underscore the gap between centrally defined expectations and the operational realities faced by health workers in PHC settings.

Overall, institutional and systemic barriers—ranging from excessive programmatic demands and inadequate digital infrastructure to poor inter-sectoral coordination and unrealistic monitoring systems—represent critical obstacles to achieving sustainable mental health integration in primary care. Addressing these challenges requires context-based reforms that streamline workloads, enhance data systems, promote cross-sector collaboration, and align performance indicators with realistic operational capacities.

## Discussion

The integration of mental health services into primary health care (PHC) is widely recognized as a fundamental strategy for advancing health equity in low- and middle-income countries (LMICs) [35, 36]. In Iran, despite the nationwide implementation of mental health programs through the public health network, evidence suggests a substantial gap between planning and effective execution. This qualitative study identified four key domains of implementation barriers: limited human capacity, inadequate infrastructure, socio-cultural obstacles, and institutional-systemic challenges. The discussion below critically interprets these findings in the context of national and international evidence, emphasizing their implications for policy and practice.

### Limited human capacity and professional interactions

The findings indicated that weak collaboration between general practitioners, mental health workers, and psychologists constitutes a major barrier to service delivery. General practitioners were often reluctant to document psychiatric diagnoses, thereby shifting the burden of diagnosis and clinical decision-making to non-specialist staff. This lack of role clarity disrupted referral and follow-up processes and contributed to professional demotivation. Similar challenges have been reported in studies from Nigeria [37], Pakistan [38], and Brazil [39], highlighting how unclear professional boundaries can lead to fragmented care. In the Iranian PHC system, this phenomenon reflects a broader structural issue—namely, the lack of interdisciplinary coordination mechanisms and limited supervision frameworks that could promote shared responsibility among health professionals.

In addition to interactional barriers, insufficient training for newly recruited staff negatively affected clinical performance and service quality. As reported by Kakuma et al. [12] and the WHO [40], in many resource-limited settings, in-service training on mental health is either nonexistent or lacks formal evaluation. These observations resonate with our findings, particularly regarding contract workers who enter the system without standardized preparation. In the Iranian context, continuous education is often constrained by workload and resource limitations, resulting in uneven skill levels among PHC personnel. Developing structured mentorship and on-the-job training—similar to what has been implemented in “core competency” programs for disaster risk management among Iranian nurses—could enhance the sustainability and quality of mental health care delivery.

Moreover, excessive workload caused by the integration of multiple programs into single service units has led to burnout, job dissatisfaction, and reduced quality of psychological interventions. Shanafelt et al. [41] and the OECD [42] refer to this phenomenon as “multi-tasking fatigue,” which is prevalent in many healthcare systems. In Iran, where health workers simultaneously manage vaccination, chronic disease screening, nutrition counseling, and traditional medicine programs, mental health responsibilities often become secondary. These findings suggest that beyond human resource shortages, there is an urgent need to rationalize workloads and strengthen administrative support mechanisms to maintain the quality and sustainability of PHC-based mental health services.

### Inadequate physical and technological infrastructure

Mental health service delivery relies heavily on privacy, psychological safety, and effective communication tools. However, in many centers examined, consultations occurred in open or shared spaces, lacking confidentiality. Studies by Patel in India [43] and Gureje in Nigeria [44] similarly demonstrate that the absence of dedicated counseling rooms is associated with reduced patient trust and early discontinuation of treatment. The Iranian experience reflects how infrastructural inadequacies can undermine patient comfort and provider efficiency. Investments in physical space and supportive environments should thus be viewed as integral—not auxiliary—components of health system reform.

From a technological standpoint, the removal of functionalities such as automated SMS reminders in systems like SIB and the lack of online access to patient records disrupted continuity of care. Comparable findings have been reported in Kenya [45], Afghanistan [46], and Zambia [17], where access to simple technologies like tablets and telephones improved follow-up rates. In Iran, restoring and upgrading digital tools can directly enhance communication and follow-up efficiency. Likewise, the physical design of health centers—often unfriendly to elderly or disabled clients [47]—indicates that inclusive design principles should be integrated into PHC infrastructure planning. Overall, addressing these shortcomings requires both infrastructural investment and system-level recognition of the environment as a determinant of mental health service quality.

### Cultural and social barriers

The study revealed that stigma, lack of awareness, and family resistance remain dominant social barriers to mental health service utilization. Fear of labeling and social judgment prevents many individuals from acknowledging their psychological needs, particularly in smaller communities where gossip and moral scrutiny are pervasive. These findings align with global evidence indicating that stigma remains one of the most persistent challenges to improving mental health [48–50]. In the Iranian context, stigma often intersects with cultural and religious norms that equate mental illness with personal weakness or spiritual failure. Consequently, stigma reduction must be pursued through culturally grounded strategies—such as public education campaigns involving religious leaders and community influencers—to normalize help-seeking behaviors and promote social acceptance of mental health services. Similar barriers to family engagement in mental health care have also been documented in Iranian qualitative research, which highlights communication difficulties and limited policy support for family-centered approaches [51].

Public unawareness of available mental health services further compounds this problem. Many citizens remain unaware that PHC centers provide free psychological counseling, leading them to seek costly private services instead. Studies from Egypt [52] and India [53] have reported similar patterns of misinformation and avoidance. Addressing this gap requires coordinated communication strategies, including social media engagement, SMS campaigns, and community outreach, to enhance mental health literacy. The lack of cooperation from families—especially in cases involving adolescents and women—was also notable. A study from Pakistan [54] reported similar parental resistance, underscoring the need for family-centered education and participatory approaches. Future initiatives could build on evidence from Iranian studies promoting family involvement in chronic mental illness management, adapting similar frameworks for PHC-level interventions.

### Institutional and systemic challenges

The structure of mental health service delivery in Iran remains fragmented, characterized by overlapping responsibilities and weak monitoring mechanisms. Unrealistic performance indicators—such as fixed quotas for consultations or screenings—create administrative burdens that discourage staff and compromise data quality. The OECD has warned that such *“context-blind”* indicators can undermine service quality [55]. The findings of this study underscore the need for context-sensitive metrics that are tailored to the population size and operational capacity of each health center. Comparable organizational and monitoring challenges have been reported in other Iranian qualitative studies on hospital management and crisis preparedness, where limited coordination and over-centralized control were found to hinder effective implementation [25].

Furthermore, incompatibility between digital platforms such as SIB and clinical protocols continues to disrupt information flow and reduce follow-up efficiency. The WHO’s *Mental Health Atlas* [56] similarly emphasizes the importance of harmonizing health information systems with clinical workflows—an approach urgently required within Iran’s PHC network. Weak inter-sectoral collaboration with educational institutions and hospitals also emerged as a systemic gap, echoing findings from Brazil [27] and Turkey [57]. In the absence of effective two-way referral and coordination mechanisms, the continuum of care for individuals experiencing crises—such as psychosis or suicidal behavior—remains severely interrupted.

Finally, the lack of bottom-up policy feedback, as highlighted by participants and corroborated by evidence from Lebanon [58], reflects a broader governance issue: mental health policies are frequently formulated at the national level without meaningful input from frontline practitioners. Developing participatory policymaking structures that incorporate field-level experiences could improve policy realism, enhance accountability, and foster long-term ownership of mental health programs within the PHC system.

### Policy implications and recommendations

To ensure the effective implementation of mental health programs within primary care systems, three foundational principles must be upheld: multi-level stakeholder engagement, policy realism, and sustained investment in human capital. Based on the findings of this study, we propose the following recommendations. These recommendations are aligned with recent Iranian studies on guideline adaptation and family-centered collaborative care for chronic mental illness, which emphasize the importance of interdisciplinary teamwork, local adaptation of evidence-based guidelines, and culturally sensitive implementation approaches [59–61].

1. Revise both formal and informal training programs for mental health personnel to strengthen clinical and communication competencies.
2. Design safe, private, and dedicated counseling spaces within PHC centers to ensure confidentiality and psychological comfort for clients.
3. Launch comprehensive anti-stigma campaigns in collaboration with local leaders, media outlets, and community influencers.
4. Redesign performance indicators based on contextual feasibility and the operational capacity of regional health centers.
5. Integrate mental health information into the national digital health infrastructure through interoperable and user-friendly platforms.

These recommendations are also consistent with recent Iranian research on hospital crisis management and ethical education, which highlight the role of organizational learning, professional ethics, and cross-sectoral collaboration in building resilient and responsive health systems.

### Strengths and limitations

This study provides an in-depth exploration of implementation barriers from the perspective of PHC providers, offering contextually grounded evidence rarely captured in prior Iranian research. The use of qualitative content analysis and purposive sampling with maximum variation enhanced the richness and credibility of the findings. However, the study was limited to one province, and the sample size was modest, which may affect the transferability of results. Moreover, as interviews were conducted only with health personnel, the perspectives of patients and families were not directly included. These limitations highlight opportunities for broader multi-site and multi-stakeholder research.

### Future research directions

Future studies should incorporate the perspectives of service users, caregivers, and community representatives to provide a more comprehensive understanding of implementation dynamics. Participatory and mixed-methods research can help design and evaluate interventions targeting stigma reduction, inter-sectoral collaboration, and digital health optimization. Comparative or longitudinal analyses could further assess how policy reforms and workforce capacity-building influence mental health outcomes within Iran’s evolving PHC system.

## Conclusion

This qualitative study highlights a range of systemic and structural barriers that hinder the effective implementation of mental health programs within primary health care (PHC) in southern Iran. Identified challenges—spanning governance, human resources, infrastructure, health information systems, and community engagement—underscore the need for comprehensive policy and management reforms to ensure sustainable integration of mental health into PHC services.

The findings clearly suggest that without improving intersectoral coordination, empowering the health workforce, strengthening digital and physical infrastructure, and fostering greater public awareness and acceptance of mental health services, full and lasting integration into PHC will remain elusive. The study advocates for a systems-oriented approach to mental health policymaking and recommends the development of evidence-based, context-sensitive interventions at both national and subnational levels.

Given that this study exclusively examined the perspectives of service providers and health system actors, future research should adopt participatory or mixed-methods designs to incorporate the views of patients, families, and other stakeholders. Such inclusive approaches will offer a more holistic understanding of the barriers and opportunities for scaling up mental health programs in primary care.

## Data Availability

The datasets used and/or analyzed during the current study are available from the corresponding author upon reasonable request.

## Abbreviations

PHC: Primary Health Care
WHO: World Health Organization
UHC: Universal Health Coverage
LMICs: Low- and Middle-Income Countries
mhGAP: Mental Health Gap Action Programme
SIB: Integrated Health System
OECD: Organisation for Economic Co-operation and Development
